# Host Genetic Risk Factors Associated with COVID-19 Susceptibility and Severity in Vietnamese

**DOI:** 10.3390/genes13101884

**Published:** 2022-10-18

**Authors:** Vu Phuong Nhung, Nguyen Dang Ton, Tran Thi Bich Ngoc, Ma Thi Huyen Thuong, Nguyen Thi Thanh Hai, Kim Thi Phuong Oanh, Le Thi Thu Hien, Pham Ngoc Thach, Nong Van Hai, Nguyen Hai Ha

**Affiliations:** 1 Institute of Genome Research, Vietnam Academy of Science and Technology, 18 Hoang Quoc Viet, Cau Giay, Hanoi 100000, Vietnam; 2 Faculty of Biotechnology, Graduate University of Science and Technology, Vietnam Academy of Science and Technology, 18 Hoang Quoc Viet, Cau Giay, Hanoi 100000, Vietnam; 3 National Hospital for Tropical Disease, Kim Chung, Dong Anh, Hanoi 100000, Vietnam; 4 Department of Biochemistry, Hanoi Medical University, 1 Ton That Tung, Dong Da, Hanoi 100000, Vietnam

**Keywords:** SARS-CoV-2, COVID-19, host genetic variants, ABO blood type, whole-exome sequencing

## Abstract

Since the emergence and rapid transmission of SARS-CoV-2, numerous scientific reports have searched for the association of host genetic variants with COVID-19, but the data are mostly acquired from Europe. In the current work, we explored the link between host genes (SARS-CoV-2 entry and immune system related to COVID-19 sensitivity/severity) and ABO blood types with COVID-19 from whole-exome data of 200 COVID-19 patients and 100 controls in Vietnam. The O blood type was found to be a protective factor that weakens the worst outcomes of infected individuals. For SARS-CoV-2 susceptibility, rs2229207 (TC genotype, allele C) and rs17860118 (allele T) of *IFNAR2* increased the risk of infection, but rs139940581 (CT genotype, allele T) of *SLC6A20* reduced virus sensitivity. For COVID-19 progress, the frequencies of rs4622692 (TG genotype) and rs1048610 (TC genotype) of *ADAM17* were significantly higher in the moderate group than in the severe/fatal group. The variant rs12329760 (AA genotype) of *TMPRSS2* was significantly associated with asymptomatic/mild symptoms. Additionally, rs2304255 (CT genotype, allele T) of *TYK2* and rs2277735 (AG genotype) of *DPP9* were associated with severe/fatal outcomes. Studies on different populations will give better insights into the pathogenesis, which is ethnic-dependent, and thus decipher the genetic factor’s contribution to mechanisms that predispose people to being more vulnerable to COVID-19.

## 1. Introduction

Coronavirus disease (COVID-19) was caused by the severe acute respiratory syndrome-Coronavirus-2 (SARS-CoV-2). This has led to the most severe pandemic spreading over the world, affecting over 319 million people and causing over 6 million deaths globally (covid19.who.int). In Vietnam, from January 2020 to May 2022, there have been 10,698,180 confirmed cases with 43,067 deaths reported to the World Health Organization. Individuals infected with SARS-CoV-2 presented wide clinical manifestations from being asymptomatic to severe or even fatal due to acute respiratory syndromes. In the last two years, considering broad differences in the clinical symptoms of SARS-CoV-2-infected individuals, identifying the risk factors relevant to COVID-19 clinical features has been raised as a primary concern among scientists. It was reported that host factors including advanced age, the male sex, as well as comorbidities such as hypertension and diabetes, are associated with the severity and mortality of the disease. Furthermore, the hypothesis on the association between viral load and COVID-19 severity was also raised as an important matter, which was still inconclusive [1]. However, the influence of these factors could not fully explain the broad range of clinical outcomes of COVID-19 patients. Therefore, numerous studies have searched for host genetic risk factors of COVID-19. Simulations, docking [2,3], and in silico prediction [4], as well as the genome wide association study (GWAS), have been performed mostly in various European populations [5,6,7], revealing that host genetics could also contribute to COVID-19 status variations. Genetic variants or loci that may play a protective role, increase susceptibility, or have been associated with severity and mortality have been reported. In particular, a GWAS study on Italian and Spanish patients identified two susceptible loci of severe COVID-19 including one locus on chromosome 3 with multiple genes included (*SLC6A20*, *LZFTL1*, *CCR9*, *CXCR6*, *XCR1*, *FYCO1*), and the other on chromosome 9 that defines the ABO blood types [7]. This result was later replicated by a meta-analysis from three GWAS studies [5]. Additionally, the most recent GWAS study on European, Latino, and African American patients revealed novel risk variants associated with COVID-19 severity, which were located in chromosomes 19p13.3, 12q24.13, and 21q22.1 [6]. Most recently, an updated GWAS study by COVID-19 Host Genetics Initiative additionally revealed 11 significant loci, which consisted of three genes in new loci (*SFTPD*, *MUC5B,* and *ACE2*), compared with 13 loci previously described [8]. Scientific reports in Asian populations also discovered host genetic factors related to COVID-19 infection and progression such as *ACE2* in Chinese patients [9], *ACE2* and *TMPRSS2* in Japanese patients [10], 5q32 and 12q22 in Thai patients [11], and HLA system in South Asian patients [12]. In addition to GWAS and the associated data, *TLR7* located in the X chromosome was considered a potential monogenic cause that predisposed young male patients without comorbidities to severe COVID-19 [13,14]. The most recent research from 21 cohorts across 12 countries also reported that rare, deleterious *TLR7* variants are risk factors that predisposed the severity of COVID-19 (13.1-fold increase) [15].

In this study, we performed whole-exome sequencing in Vietnamese SARS-CoV-2-infected individuals and controls who were in close contact with people with COVID-19 but were negative for SARS-CoV-2. Variants of genes responsible for the SARS-CoV-2 entry pathway, host immune responses, and ABO blood groups, as well as being correlated with COVID-19, were analyzed to investigate the association between host genetic determinants and disease susceptibility and severity. The data drawn from our work are helpful to further understand the involvement of host genetic factors in the COVID-19 pathogenesis of Asian populations, which have been studied less than European ethnicities.

## 2. Materials and Methods

### 2.1. Subjects Collection and Clinical Classification

In order to suppress the COVID-19 pandemic in Vietnam during the period from 2019 to 2021, the policy deployed a very strict quarantine at medical facilities for people who were positive for SARS-CoV-2 as well as those in close contact with infected individuals. All patients were admitted to the National Hospital of Tropical Diseases from July 2020 to June 2021. The diagnosis of COVID-19 was determined by the quantitative PCR method for the SARS-CoV-2 virus from nasopharyngeal swabs. During treatment at the hospital, they were followed up and classified into three groups according to their clinical spectrum: asymptomatic (patients positive for SARS-CoV-2 but showing no symptoms) or mild (patients positive for SARS-CoV-2 and without any shortness of breath, dyspnea, or abnormal chest imaging), moderate (patients with lower respiratory tract inflammation but SpO2 > 94%), severe (patients who required admission to the intensive care unit and were supported with oxygen therapy) or fatal (patients who died due to COVID-19). The clinical manifestations were categorized according to the guidelines for diagnosis and treatment of COVID-19 of the Ministry of Health, Vietnam. All control individuals were unrelated individuals that had a history of close contact with COVID-19 patients during their infection. Additionally, the control groups had never been infected with SARS-CoV-2 before (according to the medical health declaration). They were followed up in two weeks and confirmed as SARS-CoV-2-negative by quantitative PCR twice in the concentrated isolation areas.

The informed consent forms were signed and provided by all participants, in which all essential information that the enrolled subjects needed to make the decision to volunteer for the study was prepared. This work obtained ethical approval from the Institutional Review Board of the Institute of Genome Research (No: 4-2020/NCHG-HĐĐĐ).

### 2.2. Genomic DNA Preparation

For all patients and volunteers, 2 mL of total blood was collected in EDTA containing tube and stored at −20 °C. Total DNA was subsequently extracted from 200 μL of peripheral blood by an Exgene Blood^TM^ SV kit following the manufacturer’s protocol (GeneAll, Seoul, Korea). The concentration of genomic DNA was later determined by a Qubit dsDNA BR Assay kit (ThermoFisher Scientific, Waltham, MA, USA).

### 2.3. Whole-Exome Sequencing

The concentration of total DNA was then determined by a Qubit dsDNA BR Assay kit (ThermoFisher Scientific, Waltham, MA, USA). Library constructions from the affected child were performed by Sure Select V6-Post according to the manufacturer’s protocol (Agilent Technologies, Santa Clara, CA, USA). The sequencing library was prepared by random fragmentation of the DNA, followed by 5′ and 3′ adapter ligation. Fragments ligated with an adapter were subsequently amplified by PCR and gel-purified. The enriched library was quantified using a Qubit dsDNA HS Assay Kit (Thermo Fisher Scientific, Waltham, MA, USA). DNA fragment distribution was confirmed by 2100 Bioanalyzers using a High Sensitivity DNA chip (Agilent Technologies, Santa Clara, CA, USA) with an expected size range from 200 bp to 400 bp. Paired-end sequencing was carried out on the NovaSeq platform (Illumina, San Diego, CA, USA) following the manufacturer’s instructions. The mean exome coverage was more than 100×, and each target base had at least 20× coverage. Candidate genes were selected due to their involvement in the pathways that essential for SARS-CoV-2 entry (*ACE2*, *ADAM17*, *RAB7A*, *TMPRSS2*) and immune response (*IFNAR2*, *TYK2*), or determinant for ABO blood group. Additional genes that were previously reported to be associated with COVID-19 infection/severity/hospitalization were also chosen for analysis (*DPP9*, *SLC6A20*, *LZTFL1*). SNPs of candidate genes were selected for further analysis in case their mean coverages were higher than 20× and the minor allele frequency was at least 1% in the studied population.

### 2.4. ABO Blood Type Determination

The ABO blood type of each participant was detected by genetic analysis combination of single nucleotide polymorphism in the *ABO* gene that was specific for allele O (rs8176719, c.261delG), B (rs8176746, c.796A; rs8176747, c.802C), and A (rs8176746, c.796C; rs8176747, c.802G). Two primer pairs were designed specifically for each variant of the *ABO* gene including ABO E6 for allele O and ABO E7 for alleles A and B. Primer sequences were as follows: ABO E6F: GCTGAGTGGAGTTTCCAGGT, ABO E6R: CTCAATGTCCACAGTCACTCG, ABO E7F: GGCCACCGTGTCCACTACTA, ABO E7R: CTTGTTCAGGTGGCTCTCGT. PCR reactions were performed with a final volume of 20 µL containing 10 ng total genomic DNA, 10 µL of Thermo Master Mix (ThermoFisher Scientific, Waltham, MA, USA), 0.75 μL of each primer (10 pmole/μL), and 7.5 µL of deionized water. The thermocycle was as follows: denaturation at 95 °C for 2 min, followed by 40 cycles of 95 °C for 30 s, 57 °C for 30 s, 72 °C for 30 s, and a final extension at 72 °C for 5 min. The purified PCR products were later sequenced on an ABI 3500 genetic analyzer.

### 2.5. Data Analysis

Bioedit software was used for the initial analysis of the ABO sequence. The genetic variants were determined using the reference sequence (NG_006669.2).

Gene-wide burden analysis was performed using an EPACTS package (https://genome.sph.umich.edu/wiki/EPACTS, access date on 1 January 2022) with a SKAT-O algorithm. This test was performed to investigate the risk factor of COVID susceptibility (COVID-19 patients and controls) and the clinical features (within three clinical groups). The covariances in the analysis were gender and age.

Statistical analyses were performed using the IBM SPSS statistics ver. 20. The Hardy–Weinberg equilibrium and association between categorical variables were examined using Chi-square and Fisher exact test. A one-way ANOVA test with post hoc multivariable comparisons was applied to determine the association between continuous variables. A statistical significance was considered at a *p* value < 0.05.

## 3. Results

### 3.1. General Features of the Studied Cohort

**Figure 1:**
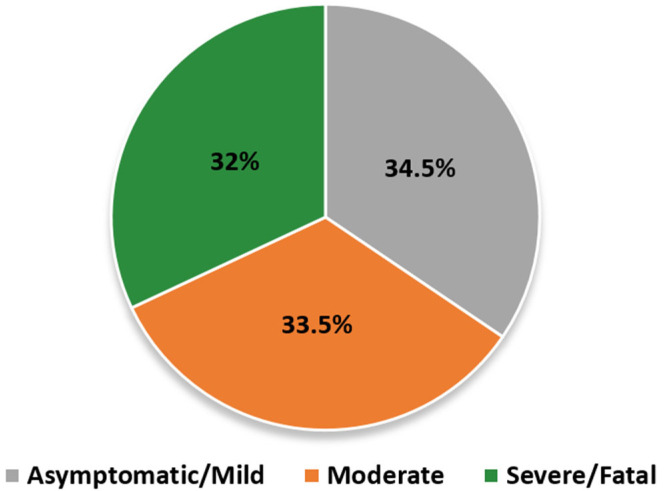
Demographic characteristics of the studied cohort. SARS-CoV-2-infected subjects were positive with quantitative RT-PCR tests. The clinical manifestations were classified into three categories: asymptomatic/mild (grey, 69 patients, 34.5%), moderate (orange, 67 patients, 33.5%), severe/fatal (green, 64 patients, 32%).

In this study, a total of 200 COVID-19 patients (89 females, 111 males) and 100 unrelated healthy (13 females, 87 males) individuals were included (Figure 1). Overall, 69 patients were clinically classified as having an asymptomatic/mild disease, 67 as having a moderate disease, and 64 as having a severe disease (15 ICU patients died). All clinical symptoms and underlying disease information of COVID-19 patients are summarized in Table 1.

The infected group showed a significantly higher frequency of females (89/200, 44.5%) than the control group (13/100, 13%) (*p* = 0, OR = 5.36) (Table 2). No association was found between gender and disease severity in the infected group. Meanwhile, within the infected subjects, patients with severe infections and those who died were significantly older than the remaining clinical groups (asymptomatic/mild infection and moderate infection) (*p* = 0). However, no difference in the mean age was found between the asymptomatic/mild infection and moderate infection individuals (*p* = 0.321). Demographic features of the studied cohort are presented in Table 2.

### 3.2. Genetic Variants Associated with the SARS-CoV-2 Susceptibility

The gene burden test resulted in no loci/SNP associated with COVID-19 susceptibility (Figure S1). Using WES data, we focused on nine genes that have been reported to be involved in the SARS-CoV-2 entry pathway (*ACE2*, *RAB7B*, *ADAM17*, *TMPRSS2*), host immune response (*IFNAR2*, *TYK2*), and/or were previously shown to be associated with COVID-19 outcomes (*DPP9*, *LZTFL1*, *SLC6A20*). All 19 SNPs of these genes showed no deviation from the Hardy–Weinberg equilibrium (HWE *p* value > 0.05, Table S3).

**Figure 2:**
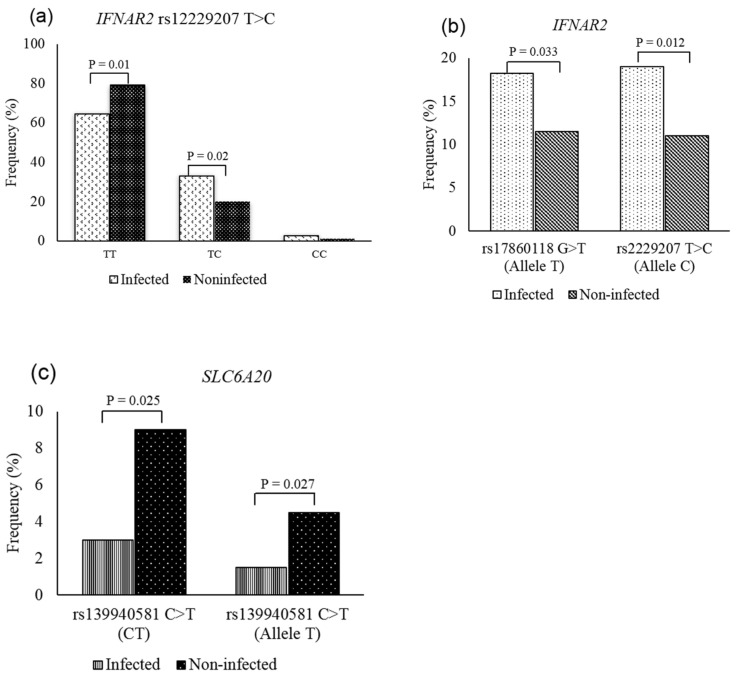
Frequency distribution of *IFNAR2* and *SLC6A20* genotypes and alleles studied cohort. (**a**) For *IFNAR2* rs12229207, COVID-19 patients carrying TT genotype showed significantly higher frequencies than the controls, while the frequency of the CT genotype was significantly higher in the controls compared with the patient’s group; (**b**) for both *IFNAR2* rs12229207 and rs17860118, the mutant alleles were significantly higher in the infected individuals compared to the controls; (**c**) for *SLC6A20* rs139940581, there was a significantly higher frequency of infected individuals carrying CT genotype and allele T compared with those observed in the controls.

Among the assessed SNPs, only 3 out of the 19 SNPs showed a significant difference between infected individuals and healthy controls, including *IFNAR2* (rs17860118, rs2229207) and *SLC6A20* (rs139940581). Specifically, for *IFNAR2*, both variants rs17860118:G>T and rs2229207:T>C (p. Phe8Ser) were significantly different between the patients and controls. The mutated alleles showed higher frequencies in the infected group, in which allele T accounted for 18.2% (*p* = 0.033, OR = 1.718) and allele C made up 19% (*p* = 0.012, OR = 1.898) (Figure 2a,b, Table S1). Regarding rs2229207:T>C, the frequency of the heterozygous genotype (TC) in the infected individuals was also significantly higher than those in the healthy subjects (*p* = 0.02). In contrast, the percentage of wild-type genotype (TT) in the infected cases was significantly lower than that in the control cases (*p* = 0.01). Among three SNPs of *SLC6A20*, the difference in frequency of rs139940581:C>T (p. Ser325Ser) between the two groups reached statistical significance. Allele T and heterozygous genotype CT comprised 1.5% and 3% in the COVID-19 patients, which were lower than the proportions detected in the controls (4.5% for allele T, 9% for CT genotype) (Figure 2c, Table S1).

### 3.3. Genetic Variants Associated with the COVID-19 Severity

**Figure 3:**
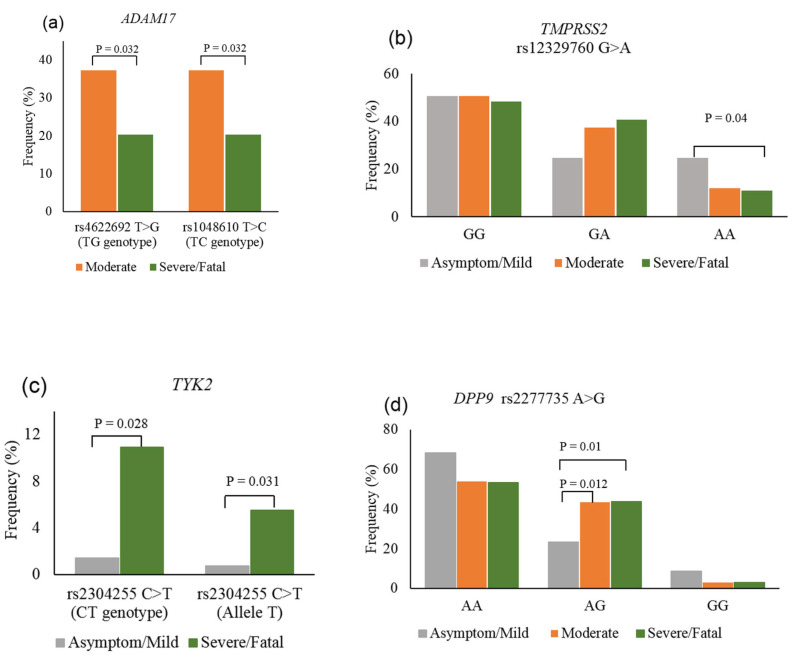
Genotype and allele frequency of *ADAM17*, *TMPRSS2*, *TYK2,* and *DPP9* in COVID-19 patients. (**a**) For *ADAM17*, the frequency of TG genotype of rs4622692 and TC genotype of rs1048610 in the moderate group were significantly higher than in the severe/fatal group, (**b**) for *TMPRSS2* rs12329760, the frequency of AA genotype in asymptomatic/mild cases was significantly higher than that in severe/fatal cases, (**c**) for *TYK2* rs2304255, CT genotype and allele T in severe/fatal cases were significantly higher than those in the asymptomatic/mild cases, (**d**) for *DPP9* rs2277735, heterozygous AG genotype showed a significantly higher frequency in moderate and severe/fatal groups compared with that in the asymptomatic/mild group.

The gene burden test resulted in no loci/SNP correlated with a severe illness of the disease (Figure S1b). Next, the correlation of host genetics with the COVID-19 outcome was examined. No statistically significant difference was determined between the compared groups in terms of variant frequencies of *IFNAR2*, *LZTFL1,* and *SLC6A20* genes, both at genotype and allele levels (*p* > 0.05) (Table S2). In the three genes involved in SARS-CoV-2 entry, the statistical analysis reached significance in the assessment of *ADAM17* and *TMPRSS2* among clinical groups. For *ADAM17*, frequencies of heterozygous genotypes TG (rs4622692:T>G) and TC (rs1048610:T>C) in the moderate cases were more significantly reported than those in the severe/fatal cases (37.3% vs. 20.3%) (Figure 3a, Table S2). For *TMPRSS2*, the assessment of rs12329760:G>A between asymptomatic/mild and severe/fatal cases resulted in statistical significance. The homozygous mutation AA frequency was found to be more common in the asymptomatic/mild group (24.6%) than in the severe/fatal group (10.9%) (*p* = 0.04, OR = 0.477) (Figure 3b, Table S2). In the two genes attributed to the host immune system, the allele T of variant rs2304255:C>T (p.Val362Phe) belonging to *TYK2* was detected to be more frequent in severe/fatal patients (5.5%) compared with that in asymptomatic/mild patients (0.7%) (*p* = 0.031, OR = 7.926). Additionally, the difference in the percentage of heterozygous CT genotypes between these two groups also reached a statistical significance, in which CT in severe/fatal cases (10.9%) was more common than that in asymptomatic/mild cases (1.4%) (*p* = 0.028, OR = 8.351) (Figure 3c, Table S2). Besides, for *DPP9* variant rsrs2277735:A>G, the percentage of heterozygous genotype AG in asymptomatic/mild infections (23.2%) was significantly lower than those observed in the moderate illness (43.3%, *p* = 0.012, OR = 0.396) and severe/fatal illness (43.8%, *p* = 0.01, OR = 0.388) (Figure 3d, Table S2).

### 3.4. Comparison of ABO Blood Group Distribution among the Enrolled Participants

After sequencing, all blood groups of 300 recruited subjects were determined, in which type O was the most common blood type detected in both patients and controls. Of the 200 infected individuals, there were 42 patients with type A (21%), 64 patients with type B (32%), 83 patients with type O (41.5%), and 11 patients with type AB (5.5%). The blood type distribution in 100 controls was 24 for type A (24%), 30 for type B (30%), 42 for type O (42%), and 4 for type AB (4%). The frequencies of four blood types were comparable between infected individuals and controls (*p* = 0.882) (Table S4).

We subsequently analyzed the association of ABO blood group distribution within the infected individuals. The statistical analysis showed no difference between the three clinical groups regarding blood group frequencies (*p* = 0.389) (Table 3). However, when the clinical groups were considered in pairs, there was a correlation between ABO blood type and disease severity. In fact, significantly higher frequencies of blood O type were observed in the asymptomatic/mild patients compared with moderate infection patients (*p* = 0.037, OR = 2.072) and severe infection/fatal patients (*p* = 0.041, OR = 2.061) (Table 3).

## 4. Discussion

In this study, the enrolled subjects were COVID-19 patients and the controls who had a history of close contact with COVID-19 patients during their infection. In fact, females accounted for only 13% of the control group, which was significantly lower than that of the infected individuals, which made up 44.5%. This indicates females are at higher risk of SARS-CoV-2 infection compared to males. Given that being male was previously reported as the highest risk factor of COVID-19 sensitivity, this disparity could be due to the factor of the male–female imbalance in the current work that resulted in the statistical bias. Due to the Vietnam government’s enforcement of strict social distancing at the early time of our project, the sample collection strategy that recruited the control group in the isolated areas is the limitation of this study.

The association of blood types with certain viral infections such as rotavirus, noroviruses, and dengue virus has been previously reported [16,17,18]. After the emergence of the COVID-19 pandemic, several studies have searched for the association of ABO blood types with the disease. All data resulted in a higher risk of COVID-19 acquiring and a viral load of the blood A type carriers, while the blood O type carriers are at a lower risk of this disease [19,20]. Additionally, a cross-sectional survey conducted in Iran showed a higher percentage of infection in the infected individuals with the AB blood type [21]. However, in the current work, we did not find any association between ABO blood types and susceptibility to SARS-CoV-2 infection. Regarding blood type and the COVID-19 clinical spectrum correlation, several reports showed that the mortality risk of group A individuals [22] and group B individuals [23] was significantly higher than group O individuals. Interestingly, in this study, patients belonging to different clinical manifestations of COVID-19 showed a significant difference in ABO blood type frequencies. We found that the patients with blood O type were at a lower risk of moderate illness or severe illness/fatal, suggesting a protective effect of blood group O. However, there was no significant correlation of blood A or B type with COVID-19 severity observed in our study. The protective role of blood O type against SARS-CoV-2, as well as several virus infections, was previously explained as the presence of both antibodies A and B in serum. The ability of these antibodies that can naturalize viruses by blocking their interaction with ACE2 receptors on the host cell membrane helps prevent the virus infection rate [22,24]. This could also be involved in the mechanism that blood O type plays as an important factor that protects COVID-19 patients from poor prognosis. Additionally, the variable observation of blood type in association with SARS-CoV-2 infection and the disease outcome could be attributed to the number of asymptomatic controls not tested for SARS-CoV-2, the population specificity, and the unknown pathogenicity of virus strains. Another retrospective study on a larger cohort will further confirm the relationship of the ABO blood type with COVID-19 in Vietnam.

*ACE2*, *ADAM17,* and *TMPRSS2* are the most well-known genes that provide instructions for making proteins essential for SARS-CoV-2 entry into the host cell. Among these genes, ACE2 variants were rare in the study cohort, while the remaining two showed more variations. ADAM17 and TMPRSS2 both act on ACE2 in a fine-tuned process and the relevant effects may be opposite in terms of ACE2 shedding [25,26]. While TMPRSS2 cleaves both ACE2 and the S protein, leading to the cellular uptake of the virus, the cleavage of ACE2 by ADAM17 resulted in the receptor shedding into the extracellular space. However, in contrast to these findings, recent works proved that ADAM17 mRNA and protein were both elevated in severe COVID-19 patients [27], and ADAM17 inhibition meliorated COVID-19-related lung inflammation [28]. Both rs4622692:T>G and rs1048610:T>C (p.S608S) of *ADAM17* had not been reported elsewhere concerning COVID-19. Our data show a significantly higher percentage of TG (rs4622692:T>G) and TC (rs1048610:T>C) in the moderate illness group compared with the severe illness/fatal group. This result suggested a protective effect of these heterozygous genotypes in reducing the likelihood of the worst COVID-19 condition. According to the discrepancy of the ADAM17 effect on the virus entry mechanism mentioned above, the exact consequence of *ADAM17* genetic variants detected in this work needs to be further deciphered.

For *TMPRSS2*, the initial examination on rs12329760:G>A (p.V197M) revealed an association of the AA genotype with a significantly higher Ct value in comparison with the GG genotype, indicating that the homozygous mutation is likely related to a lower viral load [29]. Additionally, the GG genotype tends to relate to the disease severity, but the statistical data did not reach significance [29]. The Ct value in our study was not available for all subjects, thereby we were not able to inspect the relationship between viral load and *TMPRSS2* genetic variants. Noticeably, only one out of four variants of rs12329760:G>A (p.V197M) showed a relationship with the severity. The proportion of homozygous mutation AA was significantly higher in asymptomatic/mild patients compared with severe illnesses and those who died because of COVID-19. We postulate that the AA genotype thereby possibly lessens the risk of patients being severe in respect of COVID-19 progression. The p.V197M substitution was considered deleterious by Polyphen-2, SIFT, and SNAP-2 [30]. Computation analysis further predicted that the largest pocket protein upon V197M replacement is prone to affect the protein structure [30], and amino acid substitution decreases protein stability [31]. Our findings are consistent with previous works on the correlation between *TMPRSS2* polymorphism and COVID-19 infection and severity. In the Chinese cohort, the minor allele frequency of rs12329760:G>A (p.V197M) decreased in the severe patients compared with the mild, but the difference did not reach statistical significance [32]. In Italy, a lower percentage of p.V197M minor allele was observed in the COVID-19 patients compared with the Europe reference database [33]. Owing to the importance of TMPRSS2 in virus invasion, genetic variants influencing protein structure and stability could impair its role in SARS-CoV-2 entry and lead to a wide spectrum of clinical outcomes. Further in vitro or in vivo models of SARS-CoV-2 infection are indispensable in order to prove the mechanism by which p.V197M impacts viral entry into host cells.

A GWAS study using genetic data from 2244 critically ill COVID-19 patients from UK ICUs has recently identified *IFNAR2* and *TYK2* as candidate genes significantly associated with severe COVID-19 [6]. Both IFNAR2 and TYK2 are involved in signal transduction of the type I IFN pathway by activating the STAT pathway, which plays a crucial role in the early phase of antiviral defense. A study of 50 COVID-19 patients with impaired type I IFN activity suggested that IFN type I deficiency may be indicative of critical COVID-19 critical [34]. For *IFNAR2*, we determined a relationship between two variants rs17860118:G>T and rs2229207:T>C (p.F8S) with SARS-CoV-2 infection. The higher percentage of variant alleles (T and C) of these polymorphisms in the infected individuals suggested they are COVID-19-sensitivity risk factors. Regarding critical illness, one out of three variants of *TYK2* rs2304255:C>T (p.V362F) showed a significant difference between asymptomatic/mild cases and severe/fatal cases. Notably, both T allele carriers and T allele were more frequently found in severe/fatal cases and are probably COVID-19-severity risk factors. However, findings from numerous association studies resulted in a significant association of innate immune system-related gene variants with critical COVID-19, and none of those mentioned above detected *IFNAR2* and *TYK2* variants [5,6]. For *IFNAR2*, the minor variant of rs2229207:T>C (p.F8S) showed a lower level of IFNγ [35], and this might reasonably implicate the susceptibility of minor allele carriers resulting from our study. If that were the case, the function of TYK2 would also be reduced due to the inhibition of type I IFN signal transduction and thereby facilitate viral amplification. In silico analysis by both Polyphen-2 and SIFT predicted rs2229207:T>C and rs2304255:C>T to be benign, suggesting no detrimental effect of these nonsynonymous variants.

In addition to ABO blood type, receptors, and immune genes, GWAS studies on COVID-19 have revealed the association of *SLC6A20* (rs2271616), *LZTFL1* (rs10490770, rs11385942, rs73064425), and *DPP9* (rs2109069) genes with respiratory failure caused by COVID-19 infection and critical illness [5,6,36]. However, none of those variants were detected in this study, which is understandable due to the genetic specificity of the population. The variant of *LZTFL1* rs1129183:C>T (p.D246N) presented no correlation with COVID-19 infection or the severity of the disease. Meanwhile, for variant rs139940581:C>T of *SLC6A20*, allele T, and CT genotype were demonstrated as factors that protect people from SARS-CoV-2 infection. For variant rs2277735:A>G of *DPP9*, we also identified a significant association of the AG genotype with COVID-19 clinical variability, in which the AG genotype predisposes patients to moderate or severe/fatal cases. The *SLC6A20* gene encodes for sodium transporter that interacts with the ACE2 receptor of SARS-CoV-2 [37] and altered expression of this gene in alveolar type 2 cells appeared to impact the severity of the lungs infected with SARS-CoV-2 [38]. DPP9 expression was remarkably increased in the peripheral blood of moderate/severe COVID-19 patients compared with healthy controls [39,40]. Considering the role of DPP9 as an inflammasome inhibitor, these observations assumed homeostasis responded to the deleterious inflammation in more severe cases of COVID-19 [39]. For this reason, statistical results exhibited the higher frequency of the *DPP9* heterozygous genotype in moderate/severe/fatal individuals, suggesting the expression of this protein was increased in the blood of these groups and possibly led to hyper-inflammation. However, the reason why there is a trend of a higher percentage of wild-type and homozygous mutation genotypes in the asymptomatic/mild patients is still a matter for further investigation.

For *TMPRSS2*, *IFNAR2*, *TYK2*, *LZTFL1*, *DPP9*, and *SLC6A20*, gene clusters containing these genes are promising candidates with strong evidence replicated by numerous GWAS studies [41]. In this work, we detected the clinical significance of *IFNAR2* (2 SNPs), *SLC6A20* (1 SNP) with respect to COVID-19 susceptibility and *ADAM17* (2 SNPs), *TMPRSS2* (1 SNP), *TYK2* (1 SNP), *DPP9* (1 SNP) with respect to the severity of the disease. Except for *TMPRSS2* variant rs12329760:G>A (p.V197M), the impact of other significant variants was not confirmed or discussed elsewhere. Theoretically, intronic and synonymous variants would not lead to harmful consequences. Thus, the risk may be related to unknown causal variants in close linkage with the loci. For this reason, causative genetic factors belonging to these loci and their underlying mechanism involved in COVID-19 pathogenicity should be urgently established in future studies. With the emergence of the COVID-19 pandemic, host genetic loci affecting the infection and severity of the disease were only reported in Asian populations such as *TMEM189-UBE2V1* locus and *HLA* alleles in Chinese patients [32], rs60200309-A at *DOCK2* in Japanese patients [42], and 5q32 and 12q22 in Thai patients [11]. The data drawn in this work are worth examining further in Asia, where the association of host genetic factors and ABO blood type with COVID-19 has not been extensively investigated. Importantly, the significance of association studies was interpreted due to statistical power; thus, the evidence of host genetic risk factors from our work should be replicated on larger cohorts. On the other hand, there is an urgent need to investigate the genetic profile of young Vietnamese patients with COVID-19 without pre-existing diseases but displayed critical illness. Data from such cases/case series studies could ultimately shed light on the question regarding vital pathway signaling attributed to COVID-19 pathogenesis.

## 5. Conclusions

Our study presented, for the first time, the association between genetic factors, blood type, and COVID-19 in Vietnamese patients. We postulated that the host genetic polymorphism possibly contributed to individuals’ responses to SARS-CoV-2and thus partially explainedvast diversity in COVID-19 phenotypes. The initial evidence drawn from this work is an important attempt that will substantially aid further screening approaches in identifying people at risk of SARS-CoV-2 infection as well as the critical illness of COVID-19.

## Figures and Tables

**Table 1:** Clinical characteristics of the COVID-19 patient cohort.

Characteristics	Asymptomatic/ Mild (N = 69)	Moderate (N = 67)	Severe/Fatal (N = 64)
**Concomitant disease**			
No comorbidity	57	53	14
Diabetes	1	2	8
Hypertension	0	1	13
Dyslipidemia	0	1	0
Kidney disease	1 (Acute glomerulonephritis)	1 (Addison’s disease)	7
Cardiovascular disease	2	0	3
Lung disease	0	02 (Bronchial asthma, COPD)	1 (COPD)
Liver disease	0	0	3
Thyroid disease	0	0	
Anemia	1	0	
Cancer	0	2 (lung cancer, breast cancer)	11
**Clinical symptoms**			
Fever	4	10	42
Cough	19	26	39
Shortness of breath	1	3	60

N: Number of subjects; COPD: Chronic Obstructive Pulmonary Disease.

**Table 2:** Demographic characteristics of the studied cohort.

Character	Group	SARS-CoV-2 Infected	*p* Value	Controls	*p* Value OR (95% CI)
		N = 200		N = 100	
Gender (N)	Female	Asymptomatic/Mild (26) Moderate (33) Severe/Fatal (30)	>0.05 (a)	13	**0** (b) **5.36 (2.812–10.238)**
	Male	Asymptomatic/Mild (43) Moderate (34) Severe/Fatal (34)		87	
Age (Mean ± SD)	Asymptomatic/Mild	69 (33.64 ± 13.25): *, **	**0** (†)	¯	¯
	Moderate	67 (37.43 ± 11.75): *, ***			
	Severe/Fatal	64 (50.59 ± 15.7): **, ***			

**N**: N number of subjects, **SD**: Standard Deviation, **CI**: Confidence Interval; * Post hoc Bonferroni (*p* = 0.321) between Asymptomatic/Mild and Moderate patients; ** Post hoc Bonferroni (*p* = 0) between Asymptomatic/Mild and Severe/Fatal patients; *** Post hoc Bonferroni (*p* = 0) between Moderate and Severe/Fatal patients; †: One-way ANOVA test; (**a**) Chi-squared test between clinical groups and (**b**) Infected–control individuals; **95% CI**: 95% confidence interval.

**Table 3:** Comparison of ABO blood group between the patient cohort.

Blood Group	Asymptomatic/Mild (N = 69)	Moderate (N = 67)	Severe/Fatal (N = 64)	*p* Value
A	11	17	14	0.389
B	20	21	23	
O	36	24	23	
AB	2	5	4	
	**Asymptomatic/Mild vs. Moderate** ***p* Value** **OR (95% CI)**	**Asymptomatic/Mild vs. Severe/Fatal** ***p* Value** **OR (95% CI)**	**Moderate vs. Severe/Fatal** ***p* Value** **OR (95% CI)**	**Severe/Fatal vs. Other Conditions** ***p* Value, OR (95% CI)**
A-Non A	0.174	0.382	0.638	0.835
B-Non B	0.765	0.392	0.578	0.413
O-Non O	**0.037** **2.072 (1.041–4.122)**	**0.041** **2.061 (1.028–4.135)**	0.989	0.233
AB-Non AB	0.271	0.427	1 (*)	0.747

N: number of subjects; 95% CI: 95% confidence interval; (*) Fisher exact test.

## Data Availability

All data in this study are provided in the article and Supplementary Materials.

## Supplementary Materials

The following supporting information can be downloaded at: https://www.mdpi.com/article/10.3390/genes13101884/s1, Figure S1: Manhattan plot of gene-wide burden analysis; Table S1: Distribution of host genetic variants in the COVID-19 patients and healthy controls; Table S2: Comparison of genetic variants frequency within the COVID-19 patients; Table S3: Hardy-Weinberg equilibrium of genetic variants in the study cohort; Table S4: Comparison of ABO blood group distribution between COVID-19 patients and controls.
