# Supplementary material for: Host Genetic Risk Factors Associated with COVID-19 Susceptibility and Severity in Vietnamese
Source: Genes (Basel). 2022 Oct 18;13(10):1884. doi: 10.3390/genes13101884 (PMC9601961; doi:10.3390/genes13101884)
Supplement: Supplementary file 1 [file genes-13-01884-s001.zip › genes-1940760-supplementary.pdf]

Host genetic risk factors associated with COVID-19 susceptibility and severity in Vietnamese

Vu Phuong Nhung[1], Nguyen Dang Ton[1,2], Tran Thi Bich Ngoc[1], Ma Thi Huyen Thuong[1,2], Nguyen Thi Thanh Hai[3,4], Kim Thi Phuong Oanh[1,2], Le Thi Thu Hien[1,2], Pham Ngoc Thach[3], Nong Van Hai[1,2], Nguyen Hai Ha[1,2*]

[1] Institute of Genome Research, Vietnam Academy of Science and Technology, 18 Hoang Quoc Viet, Cau Giay, Hanoi, Vietnam.

[2] Faculty of Biotechnology, Graduate University of Science and Technology, Vietnam Academy of Science and Technology, 18 Hoang Quoc Viet, Cau Giay, Hanoi, Vietnam.

[3] National Hospital for Tropical Disease, Kim Chung, Dong Anh, Hanoi, Vietnam.

[4] Hanoi Medical University, 1 Ton That Tung, Dong Da, Hanoi, Vietnam.

*** Correspondence**: nguyenhaiha@igr.ac.vn; Tel.: +84-385-368-368.

Table S1. Distribution of host genetic variants in the COVID-19 patients and healthy controls

Gene	Variants	Region	Genotype/Allele	Patients (%) N = 200, n = 400	Controls (%) N = 100, n = 200	P value OR (95% CI)
ADAM17	rs4622692 T>G	Intron	TT	144 (72)	76 (76)	0.626
			TG	54 (27)	24 (24)	
			GG	2 (1)	0	
			T	342 (85.5)	176 (88)	0.401
			G	58 (14.5)	24 (12)	
	rs1048610 T>C p.S608S	Exon 15	TT	144 (72)	74 (74)	0.851
			TC	54 (27)	26 (26)	
			CC	2 (1)	0	
			T	342 (85.5)	174 (87)	0.616
			C	58 (14.5)	26 (13)	
	rs10495562 T>C	Intron	TT	183 (91.5)	95 (95)	0.464
			TC	15 (7.5)	5 (5)	
			CC	2 (1)	0	
			T	381 (95.2)	195 (97.5)	0.185
			C	19 (4.8)	5 (2.5)	
TMPRSS2	12329760 G>A p.V197M	Exon 6	GG	100 (50)	48 (48)	0.485
			GA	68 (34)	40 (40)	
			AA	32 (16)	12 (12)	
			G	268 (67)	136 (68)	0.806
			A	132 (33)	64 (32)	
	rs118028230 G>C	Intron	GG	168 (84)	78 (78)	0.13 (*)
			GC	31 (15.5)	19 (19)	
			CC	1 (0.5)	3 (3)	
			G	367 (91.8)	33 (82)	0.259 (*)
			C	175 (87.5)	25 (12.5)	
	rs422471 C>T	Intron	CC	97 (48.5)	38 (38)	0.161
			CT	85 (42.5)	48 (48)	
			TT	18 (9)	14 (14)	
			C	279 (69.8)	121 (30.2)	0.057
			T	124 (62)	76 (38)	
	rs75603675 C>A p.G8V	Exon 1	CC	192 (96)	92 (92)	0.19 (*)
			CA	8 (4)	7 (7)	
			AA	0	1 (1)	
			C	392 (98)	191 (95.5)	0.082
			A	8 (2)	9 (4.5)	

Table S1. Distribution of host genetic variants in the COVID-19 patients and healthy controls (continued)

Gene	Variants	Region	Genotype/Allele	Patients (%) N = 200, n = 400	Controls (%) N = 100, n = 200	P value OR (95% CI)
IFNAR2	rs17860118 G>T	Intron	GG	133 (66.5)	78 (78)	0.11 (*)
			GT	62 (31)	21 (21)	
			TT	5 (2.5)	1 (1)	
			G	327 (81.8)	177 (88.5)	0.033
			T	73 (18.2)	23 (11.5)	1.718 (1.039-2.841)
			GT+TT	67	22	0.04
			GG	133	78	1.78 (1.023-3.117)
			TT	5	1	0.67
			GG+GT	195	99	
	rs2229207 T>C p.F8S	Exon 2	TT	129 (64.5)	79 (79)	0.03 (*)
			TC	66 (33)	20 (20)	
			CC	5 (2.5)	1 (1)	
			T	324 (81)	178 (89)	0.012
			C	76 (19)	22 (11)	1.898 (1.141-3.156)
			TC+CC	71	21	0.01
			TT	129	79	2.07 (1.18-3.63)
			CC	5	1	0.667
			TT+TC	195	99	
TYK2	rs371459987 C>T	Intron	GG	194 (97)	92 (92)	0.078 (*)
			GA	6 (3)	8 (8)	
			AA	0	0	
			G	394 (98.5)	192 (96)	0.082 (*)
			A	6 (1.5)	8 (4)	
	rs55882956 G>A p.R703W	Exon 15	GG	186 (93)	96 (96)	0.302
			GA	14 (7)	4 (4)	
			AA	0	0	
			G	386 (96.5)	196 (98)	0.31
			A	14 (3.5)	4 (2)	
	rs2304255 C>T p.V362F	Exon 8	CC	186 (93)	90 (90)	0.367
			CT	14 (7)	10 (10)	
			TT	0	0	
			C	386 (96.5)	190 (95)	0.377
			T	14 (3.5)	10 (5)	

Table S1. Distribution of host genetic variants in the COVID-19 patients and healthy controls (continued)

Gene	Variants	Region	Genotype/Allele	Patients (%) N = 200, n = 400	Controls (%) N = 100, n = 200	P value OR (95% CI)
DPP9	rs187026346 C>A p.G608G	Exon 16	CC	189 (94.5)	93 (93)	0.455
			CA	11 (5.5)	6 (6)	
			AA	0	1 (1)	
			C	389 (97.2)	192 (96)	0.41
			A	11 (2.8)	8 (4)	
	rs57034092 G>T	Intron	GG	170 (85)	84 (84)	1 (*)
			GT	27 (13.5)	14 (14)	
			TT	3 (1.5)	2 (2)	
			G	367 (91.8)	182 (91)	0.756
			T	33 (8.2)	18 (9)	
DPP9	rs2277735 A>G	Intron	AA	117 (58.5)	64 (64)	0.527
			AG	73 (36.5)	30 (30)	
			GG	10 (5)	6 (6)	
			A	307 (76.8)	158 (79)	0.534
			G	93 (23.2)	42 (21)	
LZTFL1	rs1129183 C>T p.D246N	Exon 8	CC	188 (94)	96 (96)	0.467
			CT	12 (6)	4 (4)	
			TT	0	0	
			C	388 (97)	196 (98)	0.474
			T	12 (3)	4 (2)	
SLC6A20	rs2271615 C>T p.A9G	Exon 1	CC	177 (88.5)	81 (81)	0.078
			CT	23 (11.5)	19 (19)	
			TT	0	0	
			C	377 (94.2)	181 (93)	0.9
			T	23 (5.8)	19 (9.5)	
	rs2191027 C>T	Intron	CC	191 (95.5)	98 (98)	0.347 (*)
			CT	9 (4.5)	2 (2)	
			TT	0	0	
			C	391 (97.8)	198 (99)	0.352 (*)
			T	9 (2.2)	2 (1)	
	rs139940581 C>T p.S325S	Exon 6	CC	194 (97)	91 (91)	0.025
			CT	6 (3)	9 (9)	3.198 (1.105-9.254)
			C	394 (98.5)	191 (95.5)	0.027
			T	6 (1.5)	9 (4.5)	4.923 (1.086-8.819)

N: number of subjects, n: number of alleles, 95% CI: 95% confidence interval, (*) Fisher exact test

Table S2. Comparison of genetic variants frequency within the COVID-19 patients

Gene	Variants	Genotype Allele	Asymptomatic/ Mild (%) N = 69, n = 138	Moderate (%) N = 67, n = 134	Severe/Fatal (%) N = 64, n = 128	Asymptomatic/Mild vs Moderate P value OR (95% CI)	Asymptomatic/Mild vs Severe/Fatal P value OR (95% CI)	Moderate vs Severe/Fatal P value OR (95% CI)
ADAM17	rs4622692 T>G	TT	51 (73.9)	42 (62.7)	51 (79.7)			0.032
		TG	16 (23.2)	25 (37.3)	13 (20.3)	0.074	0.559	0.428 (0.195-
		GG	2 (2.9)	0	0			0.939)
		T	118 (85.5)	109 (81.3)	115 (89.8)	0.355	0.284	0.051
		G	20 (14.5)	25 (18.7)	13 (10.2)			
		TG+GG	-	25	13	-	-	0.032
		TT		42	51			2.33 (1.065-5.119)
		GG	-	0	0	-	-	-
		TT+TG		67	64			
	rs1048610 T>C p.S608S	TT	51 (73.9)	42 (47.6)	51 (79.7)			0.032
		TC	16 (23.2)	25 (37.3)	13 (20.3)	0.074	0.559 (*)	0.428 (0.195-
		CC	2 (2.9)	0	0			0.939)
		T	118 (85.5)	109 (81.3)	115 (89.8)	0.355	0.284	0.051
		C	20 (14.5)	25 (18.7)	13 (10.2)			
		TC+CC	-	25	13	-	-	0.032
		TT		42	51			2.33 (1.065-5.119)
		CC	-	0	0	-	-	-
		TT+TC		67	64			
	rs10495562 T>C	TT	63 (91.3)	59 (88.1)	61 (95.3)			
		TC	4 (5.8)	8 (11.9)	3 (5.4)	0.218	0.631	0.455 (*)
		CC	2 (2.9)	0	0			
		T	130 (94.2)	126 (94)	125 (97.7)	0.952	0.143	0.121
		C	8 (5.8)	8 (6)	3 (2.3)			
TMPRSS2	12329760 G>A p.V197M	GG	35 (50.7)	34 (50.7)	31 (48.4)			
		GA	17 (24.6)	25 (37.3)	26 (40.6)	0.093	0.047	0.925
		AA	17 (24.6)	8 (11.9)	7 (10.9)			
		G	87 (63)	93 (69.4)	88 (68.8)	0.268	0.327	0.909
		A	51 (37)	41 (30.6)	40 (31.2)			

Table S2. Comparison of genetic variants frequency within the COVID-19 patients (continued)

Gene	Variants	Genotype/ Allele	Asymptomatic/ Mild (%) N = 69, n = 138	Moderate (%) N = 67, n = 134	Severe/Fatal (%) N = 64, n = 128	Asymptomatic/Mild vs Moderate P value OR (95% CI)	Asymptomatic/Mild vs Severe/Fatal P value OR (95% CI)	Moderate vs Severe/Fatal P value OR (95% CI)
TMPRSS2	12329760 G>A p.V197M	GA+AA	34	-	33	-	0.792	-
		GG	35		31			
		AA	17	-	7	-	0.04 2.98 (1.152-7.754)	-
		GG+GA	52		64			
	rs118028230 G>C	GG	54 (78.3)	57 (85.1)	57 (89.1)	0.439	0.123 (*)	0.497
		GC	14 (20.3)	10 (14.9)	7 (10.9)			
		CC	1 (1.4)	0	0			
		G	122 (88.4)	124 (92.5)	121 (94.5)	0.247	0.076	0.512
		C	16 (11.6)	10 (7.5)	7 (5.5)			
	rs422471 C>T	CC	31 (44.9)	35 (52.2)	31 (48.4)	0.563	0.919	0.713
		CT	32 (46.4)	25 (37.3)	28 (43.8)			
		TT	6 (8.7)	7 (10.4)	5 (7.8)			
		C	94 (68.1)	95 (70.9)	90 (70.3)	0.619	0.698	0.918
		T	44 (31.9)	39 (29.1)	38 (29.7)			
	rs75603675 C>A p.G8V	CC	65 (94.2)	66 (98.5)	61 (95.3)	0.167 (*)	1 (*)	0.358 (*)
		CA	4 (5.8)	1 (1.5)	3 (4.7)			
		C	134 (97.1)	133 (99.3)	125 (97.7)	0.371 (*)	1 (*)	0.361 (*)
		A	4 (2.9)	1 (0.7)	3 (2.3)			
IFNAR2	rs17860118 G>T	GG	45 (65.2)	46 (68.6)	42 (65.6)	0.36	0.227	0.857
		GT	24 (34.8)	19 (28.4)	19 (29.6)			
		TT	0	2 (3)	3 (4.8)			
		G	114 (82.6)	111 (82.8)	103 (80.5)	0.96	0.653	0.621
		T	24 (17.4)	23 (17.2)	25 (19.5)			
	rs2229207 T>C p.F8S	TT	43 (62.3)	46 (68.7)	40 (62.5)	0.206	0.232	0.685 (*)
		TC	26 (37.7)	19 (28.4)	21 (32.8)			
		CC	0	2 (2.9)	3 (4.7)			
		T	112 (81.2)	111 (82.3)	101 (78.9)	0.719	0.646	0.418
		C	26 (18.8)	23 (17.7)	27 (21.1)			

Table S2. Comparison of genetic variants frequency within the COVID-19 patients (continued)

Gene	Variants	Genotype/ Allele	Asymptomatic/ Mild (%) N = 69, n = 138	Moderate (%) N = 67, n = 134	Severe/Fatal (%) N = 64, n = 128	Asymptomatic/Mild vs Moderate P value OR (95% CI)	Asymptomatic/Mild vs Severe/Fatal P value OR (95% CI)	Moderate vs Severe/Fatal P value OR (95% CI)
TYK2	rs371459987 C>T	GG<br>GA	68 (98.6)<br>1 (1.4)	65 (97)<br>2 (2.6)	61 (95.3)<br>3 (4.7)	0.617	0.351 (*)	0.675 (*)
		G<br>A	137 (99.3)<br>1 (0.7)	132 (98.5)<br>2 (1.5)	125 (97.7)<br>3 (2.3)	0.618 (*)	0.354 (*)	0.678 (*)
	rs55882956 G>A p.R703W	GG<br>GA	63 (91.3)<br>6 (8.7)	63 (94)<br>4 (6)	60 (93.8)<br>4 (6.2)	0.745 (*)	0.746 (*)	1(*)
		G<br>A	132 (95.7)<br>6 (4.3)	130 (97)<br>4 (3)	124 (96.9)<br>4 (3.1)	0.75 (*)	0.751(*)	1 (*)
	rs2304255 C>T p.V362F	CC<br>CT	68 (98.6)<br>1 (1.4)	61 (91)<br>6 (9)	57 (89.1)<br>7 (10.9)	0.061 (*)	0.028 (*)<br>8.351 (0.998-69.896)	0.704
		C<br>T	137 (99.3)<br>1 (0.7)	128 (95.5)<br>6 (4.5)	121 (94.5)<br>7 (5.5)	0.063 (*)	0.031 (*)<br>7.926 (0.961-64.35)	0.712
		CT+TT<br>CC	1<br>68	-	7<br>57	-	0.02<br>0.119 (0.014-1)	-
		TT<br>CC+CT	0<br>69	-	0<br>64	-	-	-
DPP9	rs187026346 C>A p.G608G	CC<br>CA	64 (92.8)<br>5 (7.2)	65 (97)<br>2 (3)	60 (93.8)<br>4 (6.2)	0.441 (*)	1 (*)	0.433 (*)
		C<br>A	133 (96.4)<br>5 (3.6)	132 (98.5)<br>2 (1.5)	124 (96.9)<br>4 (3.1)	0.447 (*)	1 (*)	0.438 (*)
	rs57034092 G>T	GG<br>GT<br>TT	57 (82.6)<br>10 (14.5)<br>2 (2.9)	59 (88.1)<br>8 (11.9)<br>0	54 (84.4)<br>9 (14.1)<br>1 (1.6)	0.507	1 (*)	0.703 (*)
		G<br>T	124 (89.9)<br>14 (10.1)	126 (94)<br>8 (6)	117 (91.4)<br>11 (8.6)	0.207	0.665	0.413

Table S2. Comparison of genetic variants frequency within the COVID-19 patients (continued)

Gene	Variants	Genotype/ Allele	Asymptomatic/ Mild (%) N = 69, n = 138	Moderate (%) N = 67, n = 134	Severe/Fatal (%) N = 64, n = 128	Asymptomatic/Mild vs Moderate P value OR (95% CI)	Asymptomatic/Mild vs Severe/Fatal P value OR (95% CI)	Moderate vs Severe/Fatal P value OR (95% CI)
DPP9	rs2277735 A>G	AA	47 (68.1)	36 (53.7)	34 (53.1)	**0.028 (*)**	**0.03 (*)**	1 (*)
		AG	16 (23.2)	29 (43.3)	28 (43.8)			
		GG	6 (8.7)	2 (3)	2 (3.1)			
		A	110 (79.7)	101 (75.4)	96 (75)	0.391	0.358	0.944
		G	28 (20.3)	33 (24.6)	32 (25)			
		AG+GG	22	31	30	0.08	0.076	-
		AA	47	36	34			
		GG	6	2	2	0.16	0.177	-
		AA+AG	63	65	62			
LZTFL1	rs1129183 C>T p.D246N	CC	63 (91.3)	64 (95.5)	61 (95.3)	0.493	0.495 (*)	1 (*)
		CT	6 (8.7)	3 (4.5)	3 (4.7)			
		C	132 (95.7)	131 (97.8)	125 (97.7)	0.501	0.503 (*)	1 (*)
		T	6 (4.3)	3 (2.2)	3 (2.3)			
SLC6A20	rs2271615 C>T p.A9G	CC	60 (87)	61 (91)	56 (87.5)	0.447	0.925	0.512
		CT	9 (13)	6 (9)	8 (12.5)			
		C	129 (93.5)	128 (95.5)	120 (93.8)	0.46	0.928	0.524
		T	9 (6.5)	6 (4.5)	8 (6.2)			
	rs2191027 C>T	CC	66 (95.7)	64 (95.5)	61 (95.3)	1 (*)	1 (*)	1 (*)
		CT	3 (4.3)	3 (4.5)	3 (4.7)			
		C	135 (97.8)	131 (97.8)	125 (97.7)	1(*)	1 (*)	1 (*)
		T	3 (2.2)	3 (2.2)	3 (2.3)			
	rs139940581 C>T p.S325S	CC	67 (97.1)	67 (100)	60 (93.8)	0.496 (*)	0.427 (*)	0.054
		CT	2 (2.9)	0	4 (6.2)			
		C	136 (98.6)	134 (100)	124 (96.9)	0.498 (*)	0.432 (*)	0.056 (*)
		T	2 (1.4)	0	4 (3.1)			

N: number of subjects, n: number of alleles, 95% CI: 95% confidence interval, (*) Fisher exact test

Table S3. Hardy-Weinberg equilibrium of genetic variants in the study cohort

Gene	Variants	Region	Genotype	Observed	Expected	P HWE
ADAM17	rs4622692 T>G	Intron	TT	220	223.6	0.42
			TG	78	70.8	
			GG	2	5.6	
	rs1048610 T>C p.S608S	Exon 15	TT	218	221.88	0.42
			TC	80	72.24	
			CC	2	5.88	
	rs10495562 T>C	Intron	TT	278	276.48	0.81
			TC	20	23.04	
			CC	2	3	
TMPRSS2	12329760 G>A p.V197M	Exon 6	GG	148	136.01	0.099
			GA	108	131.97	
			AA	44	32.01	
	rs118028230 G>C	Intron	GG	246	244.8	0.91
			GC	50	52.4	
			CC	4	2.8	
	rs422471 C>T	Intron	CC	135	133.3	0.98
			CT	133	133.3	
			TT	32	33.6	
	rs75603675 C>A p.G8V	Exon 1	CC	284	283.2	0.94
			CA	15	16.5	
			AA	1	0.2	
IFNAR2	rs17860118 G>T	Intron	GG	211	211.7	0.94
			GT	83	80.6	
			TT	6	7.5	
	rs2229207 T>C p.F8S	Exon 2	TT	208	210.0	0.82
			TC	86	82.0	
			CC	6	8.0	
TYK2	rs371459987 C>T	Intron	GG	286	286.2	0.98
			GA	14	13.5	
			AA	0	0.2	
	rs55882956 G>A p.R703W	Exon 15	GG	282	282.3	0.98
			GA	18	17.5	
			AA	0	0.3	
	rs2304255 C>T p.V362F	Exon 8	CC	276	276.48	0.98
			CT	24	23.04	
			TT	0	0.3	

Table S3. Hardy-Weinberg equilibrium of genetic variants in the study cohort (continued)

Gene	Variants	Region	Genotype	Observed	Expected	P HWE
DPP9	rs187026346 C>A p.G608G	Exon 16	CC	282	281.3	0.99
			CA	17	18.4	
			AA	1	0.3	
	rs57034092 G>T	Intron	GG	254	251.2	0.45
			GT	41	46.7	
			TT	5	2.2	
	rs2277735 A>G	Intron	AA	181	180.2	0.98
			AG	103	104.6	
			GG	16	15	
LZTFL1	rs1129183 C>T p.D246N	Exon 8	CC	284	284.2	0.98
			CT	16	15.6	
			TT	0	0.2	
SLC6A20	rs2271615 C>T p.A9G	Exon 1	CC	258	259.5	0.79
			CT	42	39.1	
			TT	0	1.5	
	rs2191027 C>T	Intron	CC	289	289.1	0.97
			CT	11	10.8	
			TT	0	0.1	
	rs139940581 C>T p.S325S	Exon 6	CC	285	285.2	0.98
			CT	15	14.6	
			TT	0	0	

Table S4. Comparison of ABO blood group distribution between COVID-19 patients and controls

Blood groups	SARS-CoV-2 Infected (N = 200)	Controls (N = 100)	P value
A	42	24	0.882
B	64	30	
O	83	42	
AB	11	4	
A	42	24	0.554
Non A	158	76	
B	64	30	0.725
Non B	136	70	
O	84	42	1
Non O	116	58	
AB	11	4	0.598
Non AB	189	96	

N: number of subjects

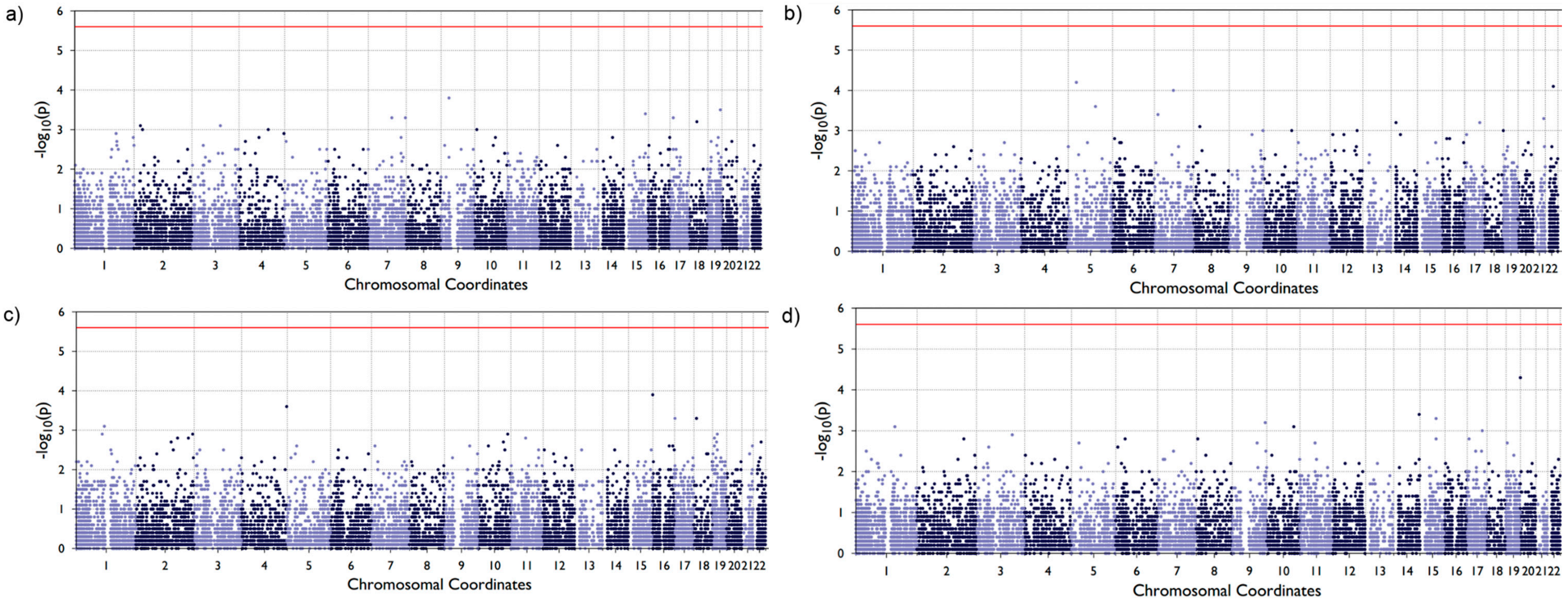

Figure S1. Manhattan plot of gene-wide burden analysis. (a) The result of COVID-19 sensitivity association (200 COVID-19 patients and 100 controls), (b) result of COVID-19 phenotype trait correlation between 69 Asymptomatic/Mild and 64 Severe/Fatal cases, (c) result of COVID-19 phenotype trait correlation between 67 Moderate and 64 Severe/Fatal cases, (d) result of COVID-19 phenotype trait correlation between 69 Asymptomatic/Mild and 67 Moderate. Observed $-\log_{10}$ values (y-axis) are displayed for all SNPs belonging to each autosomal chromosome (x-axis). The red horizon indicates gene-wide significance threshold with $P < 2.92 \times 10^{-6}$. No loci and SNP showed association with SARS-CoV-2 infection and severity of the disease.
